# *ACE2 *expression in saliva of patients with COVID-19 and its association with *Candida albicans* and *Aggregatibacter actinomycetemcomitans*

**DOI:** 10.12688/f1000research.111965.1

**Published:** 2022-05-23

**Authors:** Endang W Bachtiar, Boy M Bachtiar, Ardiana Kusumaningrum, Hari Sunarto, Yuniarti Soeroso, Benso Sulijaya, Efa Apriyanti, Citra Fragrantia Theodorea, Irandi Putra Pratomo, Yudhistira ., Defi Efendi, Fathilah Abdul Razak

**Affiliations:** 1 Department of Oral Biology, Faculty of Dentistry, Universitas Indonesia - Oral Science Research Center, Jakarta, DKI, 10430, Indonesia; 2 Department of Microbiology, Faculty of Medicine, Universitas Indonesia - Clinical Microbiology Medicine Staff Group, Universitas Indonesia Hospital, Depok, West Java, 16424, Indonesia; 3 Dental Center, Universitas Indonesia Hospital, Depok, West Java, 16424, Indonesia; 4 Department of Periodontology, Faculty of Dentistry, Universitas Indonesia, Jakarta, DKI, 10430, Indonesia; 5 Department of Pediatric Nursing, Faculty of Nursing, Universitas Indonesia and Paediatric Intensive Care Unit, Universitas Indonesia Hospital, Depok, West Java, 16424, Indonesia; 6 Department of Pulmonology and Respiratory Medicine, Faculty of Medicine, Universitas Indonesia, Jakarta, DKI, 10430, Indonesia; 7 Clinical Pathology Medicine Staff Group, Universitas Indonesia Hospital, Depok, West Java, 16424, Indonesia; 8 Department of Pediatric Nursing, Faculty of Nursing, Universitas Indonesia, and Neonatal Intensive Care Unit, Universitas Indonesia Hospital, Depok, West Java, 16424, Indonesia; 9 Department of Oral & Craniofacial Sciences, Faculty of Dentistry, University of Malaya, Kuala Lumpur, 50603, Malaysia

**Keywords:** COVID-19, ACE2, Candida albicans, Aggregatibacter actinomycetemcomitans, Fusobacterium nucleatum, Veillonella parvula

## Abstract

**Background:** A relationship between oral microbiota and susceptibility to SARS-CoV-2 infection has been extensively studied. However, the relationship between oral commensal flora and expression of angiotensin-converting enzyme 2 ( *ACE2*) remains to be established. In this observational study, we collected saliva from patients with COVID-19 and evaluated the relationship between *ACE2* expression and *Candida albicans* as well as with selected gram-negative bacteria ( *Aggregatibacter actin* *o* *mycetemcomitans*, *Fusobacterium nucleatum*, and *Veillonella parvula*). We investigated how this may be directly or indirectly involved in oral dysbiosis in patients with COVID-19.

**Methods:** We included 23 hospitalized patients admitted to Universitas Indonesia Hospital with PCR-confirmed COVID-19, with six healthy participants serving as controls. Saliva and tongue surface swabs were collected from patients with diabetes (DG) and without diabetes (NDG) and subject controls. Using quantitative PCR (qPCR) we assessed the mRNA expression of *ACE2*, the abundance of *C. albicans*, and the transcription levels of its biofilm-associated genes, agglutinin-like protein 3 ( *ALS3*), hyphal wall protein 1 ( *HWP1*), and yeast-form wall protein 1 ( *YWP1*). We also counted the relative proportion of the three selected gram-negative oral bacteria in saliva. All analyses were performed to determine the relationship between *ACE2* expression and *C. albicans* and gram-negative bacteria.

**Results:** *ACE2* mRNA expression was significantly higher in tongue swab samples than in saliva. However, no significant difference was observed between the patient groups. Conversely, DG patients had a significantly higher abundance of *C. albicans *in saliva compared to NDG patients and control group patients. The correlation and sensitivity/specificity relationship between *ACE2 *expression and *C. albicans* or the selected oral bacteria were also observed.

**Conclusions:** The data show that *ACE2* expression can be detected in saliva of patients with COVID-19 and its association with *C. albicans* and gram-negative oral bacteria might contribute toward developing an oral dysbiosis based predictor for prognosis of COVID-19 severity.

## Introduction

The oral microbiota may be involved in the pathogenesis of SARS-CoV-2 infection, the causative agent of COVID-19. In addition to other oral bacteria, *Candida* spp., particularly *C. albicans,* is a keystone commensal in the human oral cavity ^1^ that may be involved in dysbiotic events. Indeed, *C. albicans* is commonly reported for its relationships with known constituents of the oral biofilm in individuals with and without oral disease. ^2^^–^^4^ As dysbiosis of the oral microbiome has been associated with inflammatory conditions in the oral habitat of COVID-19 patients with comorbidity, as reported by Bachtiar *et al.* (preprint), ^5^ we assumed that fungal-bacterial interactions might also favor the establishment of SARS-CoV-2 infection. Therefore, our objective was to investigate the level of *C. albicans*, its pathogenicity, and to evaluate its antagonistic relationship with *Aggregatibacter actinomycetemcomitans* ^6^ in the saliva of COVID-19 patients with and without diabetes. We included *Fusobacterium nucleatum* and *Veillonella parvula* as their relationship has been previously reported. ^7^

## Methods

### Study design, patients, and specimens

The study was conducted at the Universitas Indonesia Hospital (RSUI), Depok, Indonesia. The eligible patients were recruited consecutively (up to 23), from August 2021 to September 2021. According to medical records, the patients had mild to moderate symptoms with clinically and laboratory confirmed COVID-19 infections at RSUI. Six subjects who visited the RSUI periodontal clinic served as a control. The average age of the participants was 45.1 ± 15.37 years old, and 10 patients had diabetes.

According to guidance provided by the Ethics Committee, written and oral information was given, after which written informed consent was obtained from all participants before enrolment in this study. The study protocol was approved by the ethics committee of Universitas Indonesia Hospital (protocol number: 2021/04/052). The protocol conformed to the criteria of the Declaration of Helsinki and the good clinical practical guidelines of the International Council on Harmonization, and this study was carried out in accordance with the guidelines provided by the Strengthening the Reporting of Observational Studies in Epidemiology (STROBE) statement.

Unstimulated saliva (2 ml) was collected by spitting into a sterile Falcon tube. Tongue samples were taken by swabbing the middle third of the tongue dorsum with a sterile cotton swab for a few minutes. ^8^ The obtained samples were then put into a microcentrifuge tube. All collected samples (saliva and tongue swab) were delivered promptly to the laboratory for further processing.

### Quantification of *C. albicans* by quantitative PCR

**Figure 1.**
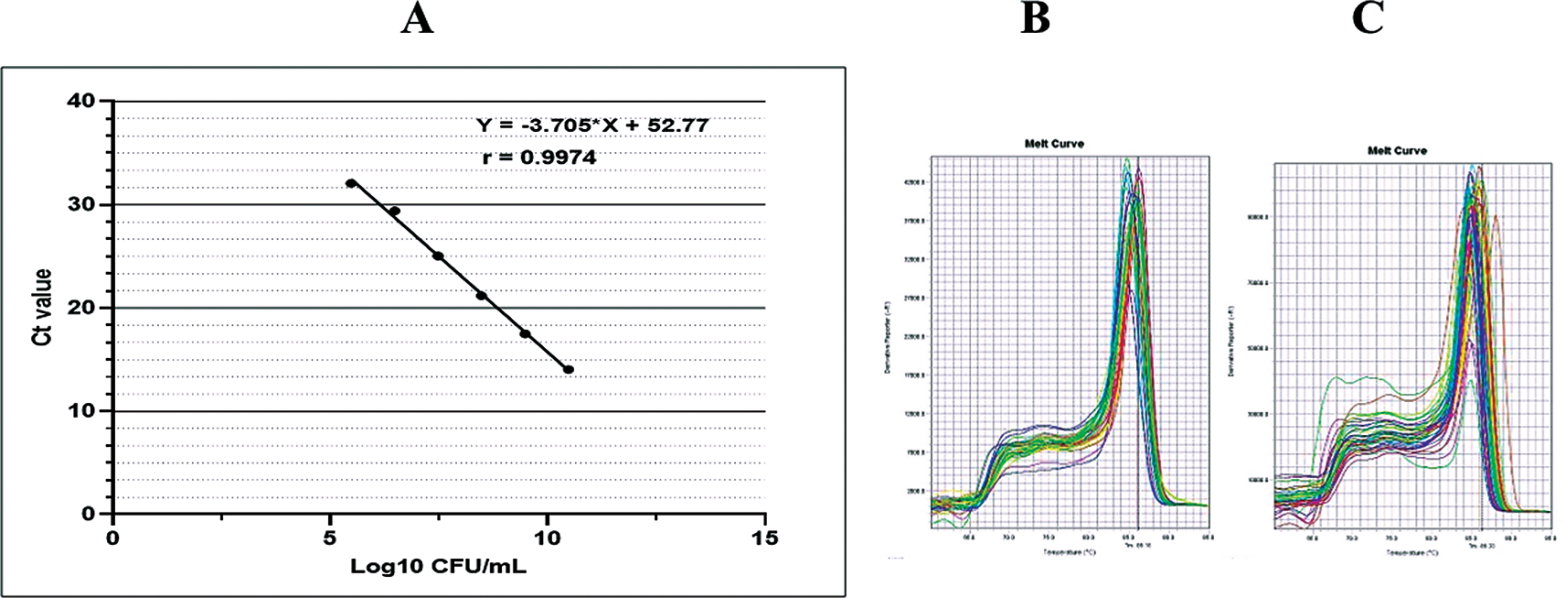
Standard curves and melt curves. Standard curve of *C. albicans* ATCC 10231 (A) and representative melt curve graph for *C. albicans* count in saliva (B) and tongue surface (C). The standard curve was linear as indicated by a linear correlation (r ^2^) of 0.9974 between the cycle threshold (Ct) value and template concentration, and a slope of -3.705.

Fungal genome extraction was performed using GENEzol™ reagents, (phenol, guanidine isothiocyanate solution) (Geneaid Biotech Ltd, New Taipei City, Taiwan), accordance with the protocol provided by the company. The concentration and quality of the obtained DNA were determined using Qubit assay reagents (Thermo Fisher Scientific, Waltham, MA, United States). To amplify DNA in saliva samples, we used quantitative PCR (qPCR) with specific primers for *C. albicans* as follows: Forward: 5′-CACGACGGAGTTTCACAAGA-3′ and Reverse: CGATGGAAGTTTGAGGCAAT-3′. ^9^ Further, the fungus amount was calculated by plotting the cycle threshold (Ct) value against the log of a standard curve shown in Figure 1A. The standard curve was constructed using a 10-fold serial dilution of DNA extracted from *C. albicans* (ATCC 10231). ^8^ The amplicon melting curves was set at 95°C for 15 seconds, 60°C for 60 secods, and 95°C for 15 seconds.

The PCR reactions were performed at a 10-minute initial denaturation at 95°C, followed by 40 cycles of denaturation at 95°C for 15-seconds annealing at 60°C for 60 second and elongation at 95°C for 15 seconds. As shown in Figure 1B and C, the qPCR products were visualized as a melting curve, that was set at 95°C for 15 seconds, 60°C for 60 seconds, and 95°C for 15 seconds.

### Proportion of *A. actinomycetemcomitans*, *F. nucleatum*, and *V. parvula* in saliva microbiota

Bacterial DNA was extracted from the saliva samples with a similar procedure described above. The levels of *A. actinomycetemcomitans*, *F. nucleatum*, and *V. parvula* colonization and the total amount of bacteria in saliva were determined using qPCR with the specific primers, ^5^ except for *A. actinomycetemcomitans*, for which we used oligonucleotides as reported elsewhere, ^10^ as follows: GTGGGGAGCAAACAGGATTAG (forward) and CCTAAGGCACAAACCCATCTC (reverse).

For both *C. albicans* and bacterial abundance, the PCR cycling process was performed in a total volume of 10 μl (comprising 5 μl of SYBR1 Selected Master Mix (Thermo Fisher Scientific, Waltham, MA, USA), DNA template (2 μl), and primer pair solution (1 μl, 300 nM/reaction). The abundance of each bacterium was determined by using the 2 ^-ΔΔCt^ method. ^11^ ΔCt was the difference between the Ct value using the primers for each bacterium and the Ct value obtained by using the primers for total bacteria in saliva. ΔΔCt was the difference between the ΔCt of the patient and control subjects, where the value of 2 ^-ΔΔCt^ shows the changes in bacterial proportion in the sample of patients relative to those of the control subjects.

### Expression of mRNA *ALS3*, *HWP1*, and *YWP1* by qPCR

For extracting total RNA, we used GENEzolTM reagent (Geneaid; Biotech Ltd, New Taipei City, Taiwan), followed by a reverse transcription kit (High-Capacity cDNA Reverse Transcription Kit, Applied Biosystems ^TM^). We followed all instructions provided by the kits. The resulting cDNA was amplified by qPCR with specific primers, as follows: *YWP1*; F: 5′-GCTACTGCTACTGGTGCTA-3′, R: 5′-AACGGTGGTTTCTTGAC-3′, *HWP1*; F: 5′-GCTCCTGCTCCTGAAATGAC-3′, R: 5′-CTGGAGCAATTGGTGAGGTT-3′, and *ALS1*; F: 5′-CAACTTGGGTTATTGAAACAAAAACA-3′, R: 5′-AGAAACAGAAACCCAAGAACAACC-3′. ^9^

Quantitative PCR analysis was performed in triplicate on an ABI StepOnePlus Real-Time PCR System with SYBR Green PCR Master Mix (Applied Biosystems). The qPCR cycling conditions consisted of a 10-minute initial denaturation at 95°C followed by 40 PCR cycles of 15 seconds at 95°C and 1 minute at 60°C. The formula of fold change 2-ΔΔCt was used to calculate the relative mRNA expression, which was compared with that of the housekeeping gene, *ACT1* with primers: F: 5′-TTTCATCTTCTGTATCAGAGGAACTTATTT-3′, R: 5′-ATGGGATGAATCATCAAACAAGAG -3′. ^12^ The formula of fold change 2 ^-∆∆Ct^ was used to calculate the relative mRNA expression of genes ( *ALS3*, *HWP1*, and *YWP1*), which was normalized to that of the housekeeping gene *ACT1.* ^13^ All values obtained from the tested patient groups were standardized and compared to the values obtained from the control subjects.

### Data analysis

In this study, we compared the amount of *C. albicans*, relative abundance of bacteria, and mRNA transcription levels of the targeted genes in two groups: patients with COVID-19 with diabetes (DG) and patients without diabetes (NDG). Statistical analyses were conducted using GraphPad Prism 9.0 (GraphPad Software, San Diego, CA, USA) (RRID:SCR_002798) (An open-access alternative is the R Stats Package 4.3.0). One way ANOVA and an unpaired Student’s t-test were used to determine the p-values between and within groups, respectively. Data are presented as the mean ± standard error (SE), and p < 0.05 was considered significant. Spearman’s correlation coefficient (r) with two-tailed p-values was used to measure the degree of association between two variables tested. The line of best fit (95% confidence interval) was shown by using linear regression. The receiver operating characteristic (ROC) method was also used to determine the sensitivity and specificity of the relationship between *C. albicans* and *A. actinomycetemcomitans* as predictors of oral dysbiosis in patients with COVID-19.

## Results

### The mRNA level of *ACE2* and the abundance of *C. albicans*

**Figure 2.**
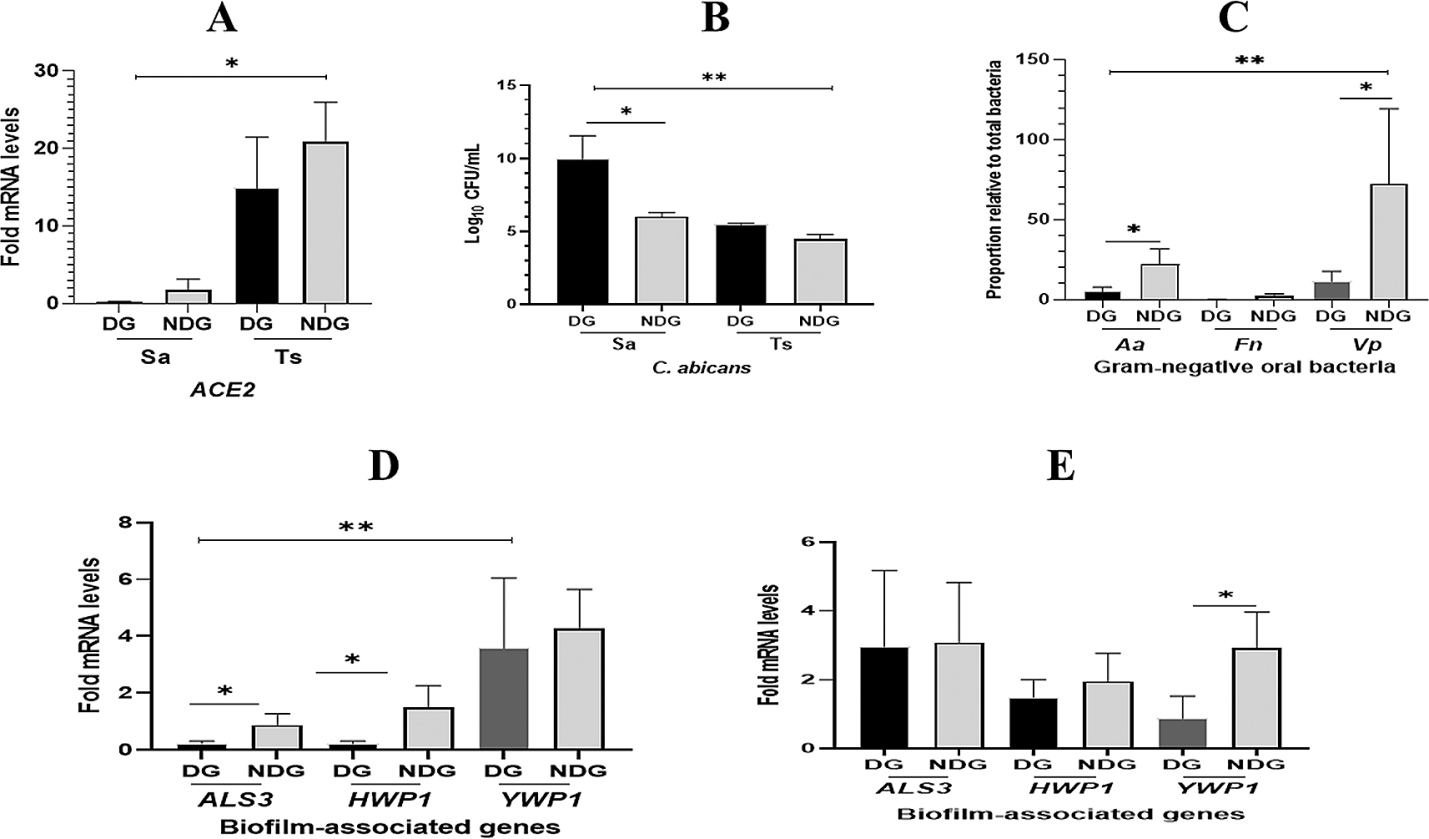
Evaluation of *ACE2* expression, the number of *C. albicans*/the proportion of selected gram-negative oral bacteria *[Aggregatibacter actinomycetemcomitans* ( *Aa*), *Fusobacterium nucleatum* ( *Fn*), and *Veillonella parvula* ( *Vp*)], and mRNA expression of selected biofilm-associated genes ( *ALS3*, *HWP1*, and *YWP1*), in different patient groups (DG and NDG) of COVID-19. In general, the transcription level of *ACE2* mRNA is higher in tongue surface (Ts) than those in saliva (Sa). Between groups, the different level of *ACE2* expression is not significant (A). The number of *C. albicans* are significantly higher (p < 0.05) in saliva than in tongue surface samples, and *C. albicans* abundance is significantly higher in DG than in NDG (B). The proportion of *Vp* is found to be the highest compared to the other two species ( *Aa* and *Fn*). Between groups, the abundance of *Aa* and *Vp* is significantly higher in DG than in NDG (C). Fold changes in gene expression detected in saliva (D) and tongue surface (E) were each compared between and within patient groups (DG and NDG). All quantitative polymerase chain reaction values were normalized according to the expression of housekeeping gene *ACT1.* All data are expressed as mean ± SE. *p < 0.05, **p < 0.001.

As shown in Figure 2A. *ACE2* mRNA expression was found in all saliva samples collected from either subject tested (DG, NDG, and control). This finding was confirmed by comparing ACE2 transcription levels on tongue surface sample (TS), where ACE2 transcription level is highly expressed in this niche. ^14^ We observed, that mean transcription level of *ACE2* detected in saliva was lower than on the TS (p < 0.05). When comparing the two group, we found that in both saliva and TS, the transcription levels of *ACE2* was higher in NDG than in DG, but the difference was not significant (p > 0.05). We further determined that *C. albicans* was also present in the saliva and TS samples collected from all subjects. In general, the count of *C. albicans* in saliva samples was higher than that of TS samples (p < 0.05). Additionally, the amount (log DNA copies) of *C. albicans* in the saliva of DG was significantly higher than that in NDG (p < 0.05). In contrast, the different number of *C. albicans* on tongue surface found in either group was not significant (p > 0.05) ( Figure 2B).

### Relative abundance of *A. actinomycetemcomitans*, *F. nucleatum*, and *V. parvula*

The qPCR results showed that the proportion of each bacterium in all subjects tested was lower in DG than in NDG. We found that the proportion of *A. actinomycetemcomitans* and *V. parvula* in NDG was >20% higher than that in DG (<10%). Subsequently, in either group tested, the abundance of *F. nucleatum* was found to be the lowest (<5%) compared to the proportion of the other two species ( Figure 2C).

### Transcription levels of *C. albicans* biofilm-associated genes

The qPCR results showed that the transcription levels of *ALS3* and *HWP1* in saliva were significantly lower than those in *YWP1* (p < 0.05) *.* We found that the transcription of both hypha-associated genes ( *ALS3* and *HWP1*) was significantly higher in NDG than in DG (p < 0.05), whereas no difference was found in the expression of *YWP1* mRNA ( Figure 2D). Furthermore, we analyzed the relative expression levels of each gene on the tongue surface (TS). As expected, in both groups tested, the hypha-related genes ( *ALS3* and *HWP1*) showed upregulation at a similar level. Conversely, a higher level of *YWP1* mRNA expression was significantly detected on the TS of NDG subjects than in DG subjects ( Figure 2E).

**Figure 3.**
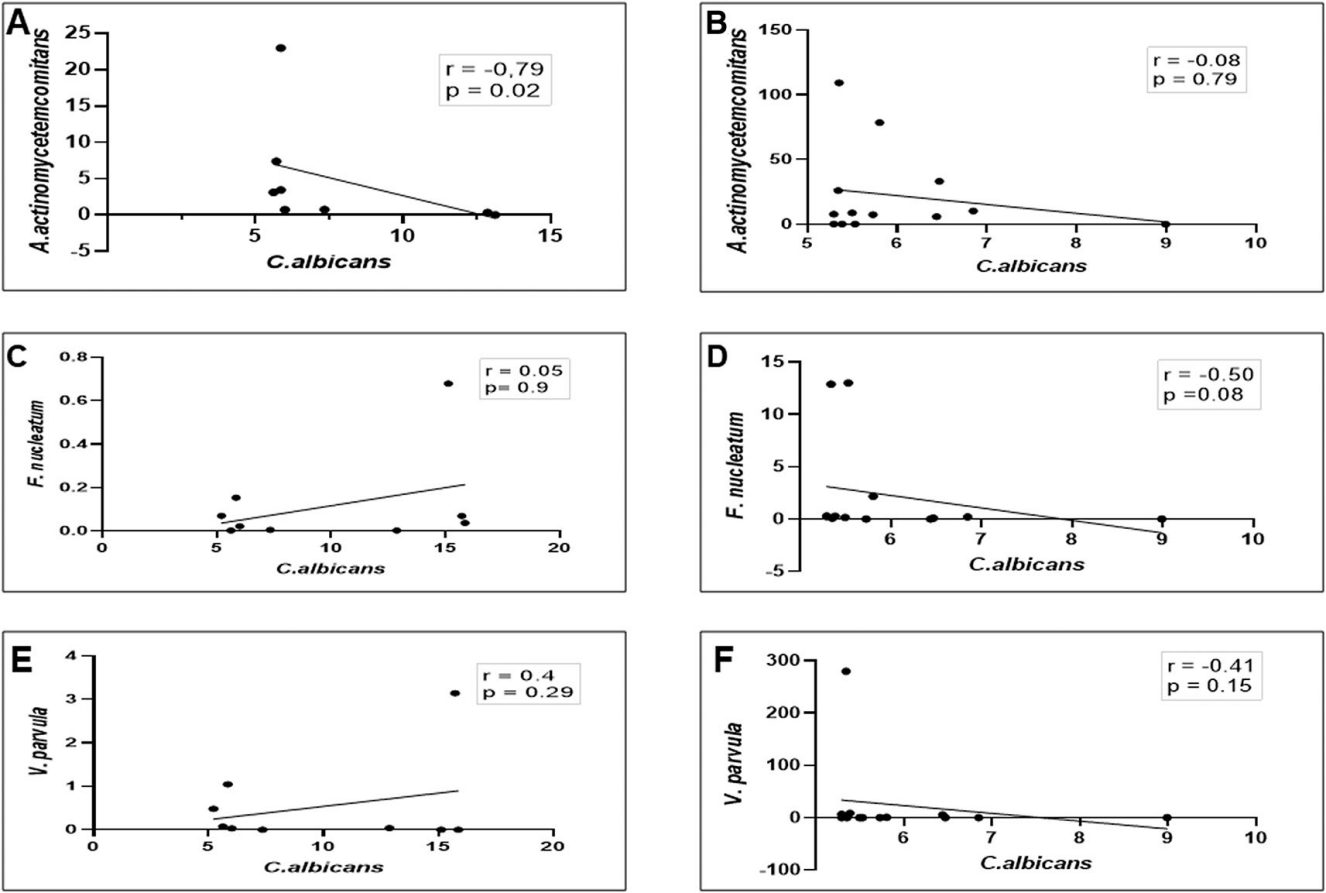
Relationship between the number (colony forming units/mL) of *C. albicans* ( *Ca*) and the three gram-negative oral bacteria *A. actinomycetemcomitans* ( *Aa*) *, F. nucleatum* ( *Fn*), and *V. parvula* ( *Vp*). In either patient group (DG or NDG), a strong negative correlation is consistently seen between *Ca* and *Aa* (A and B), while the other two bacteria show a weak positive in DG (C and E) and negative correlations in NDG, respectively (D and F). Spearman correlation coefficient (r ^2^) and exact p-values are given.

Moreover, as shown in Figures 3A and B, a strong negative linear correlation was observed between the abundance of *C. albicans* and the relative proportion of *A. actinomycetemcomitans* in DG (r = -0.79, p = 0.02), whereas in NDG, a low negative, non-significant correlation was observed between the two oral microorganisms (r = 0.08, p = 0.79). We noted, that in DG there was a positive but not significant correlation between the proportion of *C. albicans* and *F. nucleatum* (r = 0.05; p = 0.9) as well as with *V. parvula* (r = 0.24; p = 0.29) Conversely, a negative non-significant correlation was observed between *C albicans* and *F. nucleatum/V. parvula.* The correlation coefficients were r = -0.5, p = 0.08, and r = -0.41, p = 0.15, respectively ( Figure 3C– F).

**Figure 4.**
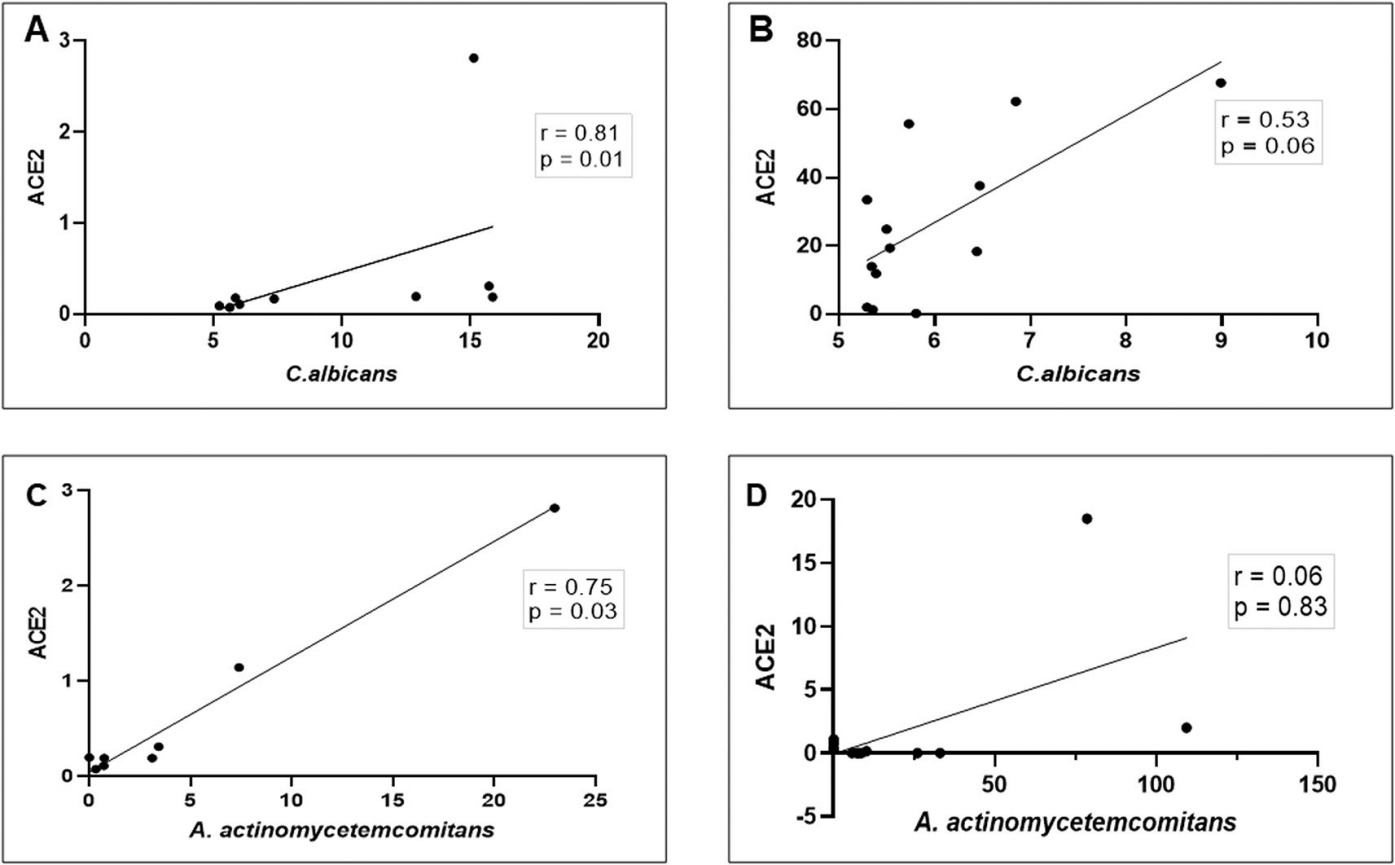
Scatter diagram illustrating the correlation between mRNA expression of *ACE2* and *C. albicans*/ *A. actinomycetemcomitans* in patients with COVID-19 with (DG) and without (NDG) diabetes. These observations indicate that in DG, the correlation between *ACE2* mRNA expression and the abundance of *C. albicans*/ *A. actinomycetemcomitans* is strongly positive (A and C), while in NDG the correlation is weakly positive (B and D). Spearman correlation coefficient (r2) and exact p-values are given.

**Figure 5.**
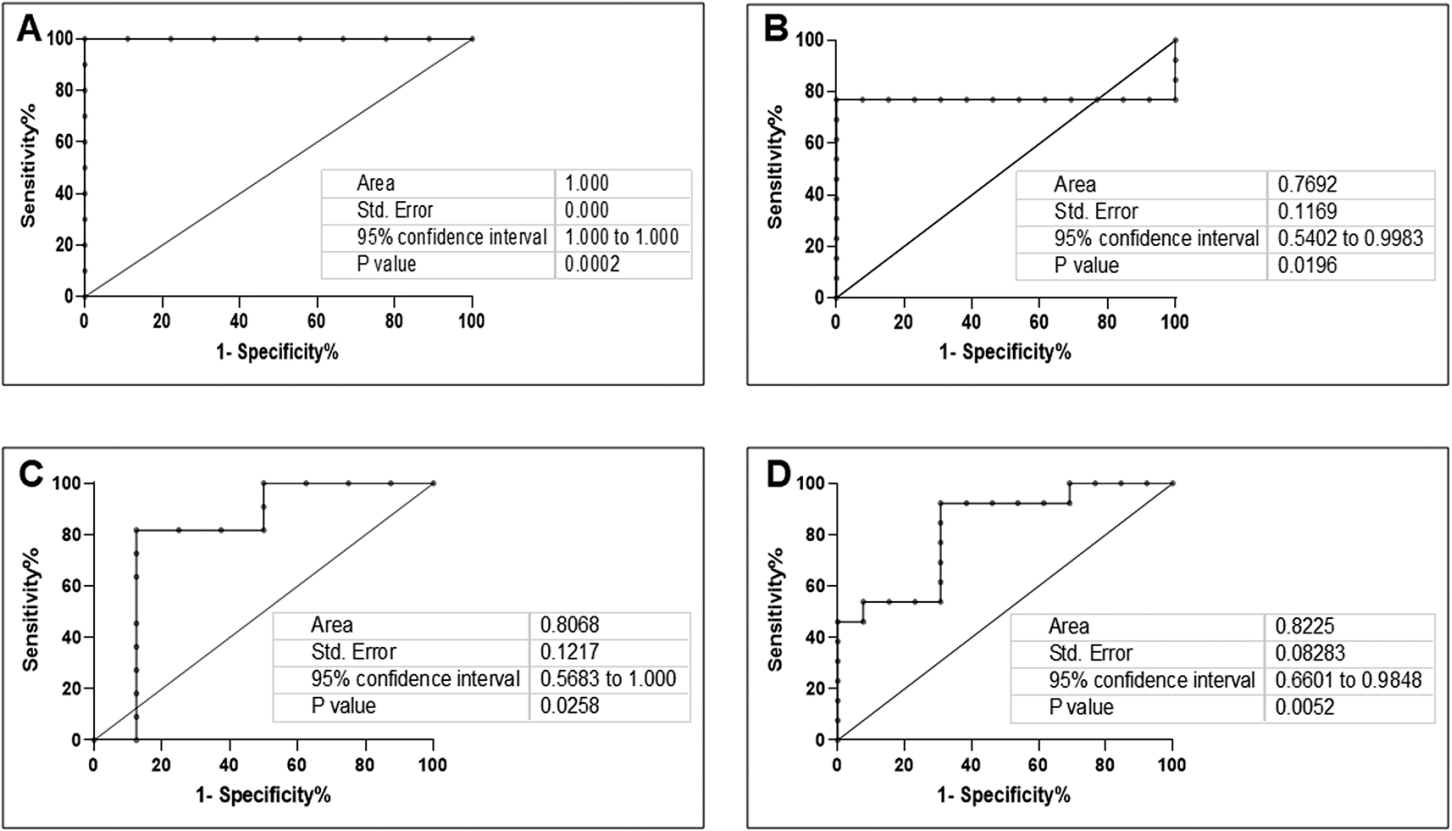
Receiver operating characteristic curve (ROC) showing the plot and the best cut-off point of the relationship between the mRNA expression of *ACE2* and *C. albicans* or *A. actinomycetemcomitans* in DG (A and C) and NDG (B and D), respectively.

As SARS-CoV-2 has been consistently detected in the saliva of infected patients, ^15^ we further examined the relationship between *ACE2* expression and the relative abundance of *C.* *albicans* and *A. actinomycetemcomitans* in the saliva of patients with COVID-19. As shown in Figure 4A– D, a strong and significant positive correlation between *ACE2* mRNA transcription and the abundance of *C. albicans/*proportion of *A. actinomycetemcomitans* was observed in DG (r = 0.81, p = 0. 01 and r = 0.75, p = 0.03, respectively). In NDG, the correlation was positive, but not statistically significant (r = 0.53, p = 0.06 and r = 0.06, p = 0.83, respectively). Based on these results, we evaluated the accuracy of the combination of *ACE2* and *C. albicans/A. actinomycetemcomitans* relationship analyses. We revealed that the area under the curve (AUC) of the *ACE2*/ *C. albicans* association, in DG was 1 (95% CI: 1 to 1, p < 0.0002; Figure 5A), and in NDG, the AUC was 0.76 (95% CI: 0.54 to 0.99, p < 0.019; Figure 5B). For the relationship between *ACE2* and *A. actinomycetemcomitans*, in DG the AUC was 0.80 (95% CI: 0.56 to 1, p < 0.02; Figure 5C). In NDG, the AUC was 0.82 (95% CI: 0.66 to 0.98, p < 0.005; Figure 5D).

## Discussion

This study found that *ACE2* expression was detected at a lower level in saliva than on the tongue surface, indicating that saliva from patients with COVID-19 might harbor epithelial cells containing SARS-CoV-2. However, the epithelium of the tongue is likely the primary target of SARS-COV-2 in the oral cavity, which is consistent with previous findings. ^16^^,^^17^ Therefore, although the oral environment is not the main target of SARS-CoV-2, the oral cavity could constitute both a portal of entry and a reservoir for the virus. This finding also indicated that *ACE2* may be crucial for the progression and prognosis of COVID-19. Hence, exploring *ACE2* expression under different physiological conditions may help predict the susceptibility of SARS-CoV-2 in different cohorts, such as COVID-19 patients with and without comorbid diabetes. The comorbidity observed in our subjects appeared to accelerate the expression of *ACE2* mRNA in oral niches. Since the susceptibility to SARS-CoV-2-driven infection correlates with *ACE2* expression, ^18^ it is possible that in our subjects, either the tongue or epithelia-containing saliva have been exposed to SARS-CoV-2 infection. We assumed that this result could be linked to the clinical status of our COVID-19 patients, which, according to the patient’s medical history, only developed mild to moderate illness. ^19^

In this study, we aimed to compare the interactions observed between *C. albicans* and gram-negative oral bacteria. As an important constituent of oral commensal flora, *C. albicans* shows a diverse inter-kingdom relationship under certain conditions, ranging from synergistic to antagonistic. ^20^^–^^22^ The results of this study showed that the number of *C. albicans* in the saliva of both patient groups was increased compared to the fungal count on the tongue surface, but a significant increase was only observed in DG. This finding indicates that although saliva provides antimicrobial activity against the opportunistic oral fungal pathogen, ^22^ this critical function is less effective in patients with COVID-19 with diabetes. Therefore, it is possible that the protective functions normally observed in saliva, such as cleansing, lubrication, and antibacterial activity, had changed in our patients with COVID-19.

Additionally, studies have shown that most patients with COVID-19 have one or more systemic (e.g., use of broad-spectrum antibiotics, use of corticosteroids, immunosuppression) or local (e.g., use of dental prostheses, reduced salivary flow due to use of medication) risk factors that favor *Candida* proliferation. ^23^^–^^25^

Previous studies have shown that *Candida* airway colonization is associated with prolonged use of mechanical ventilation and length of hospital stay. ^26^^,^^27^ According to the medical records, all patients had received a noninvasive supplemental device. However, we did not have any data regarding how long the patients had received the device at the time the samples were collected. Additionally, although the local risk factors had not been measured, the high count of salivary *C. albicans* found in this study may suggest that low salivary flow rate facilitates salivary colonization by this fungus, ^28^ as reported previously in elderly populations. ^8^ Thus, we assumed that the physiological effects of COVID-19 on salivary gland secretion ^29^^,^^30^ might affect the salivary flow rate of patients with COVID-19.

Additionally, we analyzed the pathogenicity of *C. albicans* in saliva and on the tongue surface by comparing the expression of selected biofilm-associated genes ( *ALS3, HWP1*, and *YWP1*), since these genes alters the morphology of the fungus, from the yeast to hyphal form. ^31^

Our data showed that in both patient groups (DG and NDG), the transcription levels of *ALS3* and *HWP1* were downregulated in saliva but conversely upregulated in tongue sample swabs. This finding suggests that the tongue is an ideal biotic surface in oral habitats for *C. albicans* attachment and growth. This study also revealed no difference in the pathogenicity of *C. albicans* (evaluated by qPCR) between DG and NDG patients with COVID-19. Hence, the results showed that the tongue surface is a better oral sample for detecting *C. albicans* pathogenicity in patients with COVID-19, irrespective of the diabetic condition.

*ALS3* (adhesion-related gene) and *HWP1* (hypha-specific gene) ^32^ are produced predominantly during biofilm formation, while *YWP1* is a yeast-associated gene involved in the anti-adhesive activity of *C. albicans.* ^31^ Therefore, the higher expression of *ALS3* and *HWP1* mRNAs on the tongue surface indicate that this is an important reservoir of *C. albicans* colonization. From here the fungus may dislodge into the saliva, where the expression of *YWP1* was found to be higher.

An additional phenomenon revealed in this study was the relative proportion of gram-negative bacteria in patients with COVID-19. Our data indicated that the selected oral bacteria were found in the saliva of all participants recruited in the current investigation (DG, NDG, and control). This finding suggests that the bacteria exist as normal microflora in the oral cavity, and they may have been involved in disease processes observed in our patients with COVID-19. ^5^ The current study highlighted that in the presence of *C. albicans*, the most abundant gram-negative bacteria in both diseased groups (DG and NDG) were *V. parvula*, followed by *A. actinomycetemcomitans*, while *F. nucleatum* was the least abundant. Moreover, all species can be detected in periodontitis patients with diabetes, ^33^ but only *F. nucleatum* and *V. parvula* have been reported to be positively associated with COVID-19-associated events. ^34^^,^^35^ Therefore, in this study, it was deemed pertinent to assess the relationship between the proportions of these three gram-negative bacteria and the abundance of *C. albicans* in the COVID-19 related oral environment. Analysis of saliva samples showed that in NDG (patients without diabetes), the proportion of all selected gram-negative bacteria had a significant negative correlation with the increasing load of *C. albicans* DNA *.* Interestingly, in DG (patients with diabetes), only the proportion of *A. actinomyces* was consistently and significantly negatively correlated with a higher count of *C. albicans.* This finding supports our previous work regarding the ability of *A. actinomycetemcomitans* to reduce biofilm formation by *C. albicans* when grown in mono ^36^ or dual species with *Streptococcus mutans.* ^6^ The current findings provide additional information *in vivo*, whereby the salivary component in patients with COVID-19 modulates the relationship pattern shown by *C. albicans* when interacting with *A. actinomycetemcomitans.* In the present study, a negative association between the fungus and periodontal pathogen was observed by counting both microflora in saliva samples collected from patients with COVID-19. The relationship pattern may be relevant, since it not only translates closer to the real inter-kingdom relationships *in vivo* (oral cavity), but also demonstrates the relationships between *C. albicans* and other selected gram-negative bacteria in the salivary environment. From the results here, the negative association between *C. albicans* and *A. actinomycetemcomitans* observed in salivary microbiota could be multifactorial and not be simply caused by the effect of SARS-CoV-2 in the oral cavity. However, this may explain why the presence of SARS-CoV-2 in the oral cavity favors the emergence and persistence of dysbiosis in another oral niche, including the periodontal microenvironment. ^5^ Our observations provide additional information, in which there is a strong negative correlation between the “key stone commensal” oral microflora, *C. albicans* ^1^ and periodontopathogen, *A. actinomycetemcomitans.* ^37^ Interestingly, we found that both *C. albicans* and *A. actinomycetemcomitans* showed a positive correlation with *ACE2* expression, and a strong correlation was observed in patients with COVID-19 accompanied by diabetes. Therefore, it was deemed relevant to assess these relationships by determining the sensitivity and specificity of the association. Using ROC curve analyses, we found that the antagonistic relationship between *C. albicans* and *A. actinomycetemcomitans* and its respective correlation with *ACE2* expression had an obvious effect in distinguishing between DG and NDG and could be used as a biomarker with a certain degree of accuracy. Thus, the correlation of *ACE2* expression with *C. albicans/A. actinomycetemcomitans* had a higher predictive value for oral dysbiosis in patients with COVID-19 with diabetes.

The literature shows that *C. albicans* is implicated in oral diseases, including caries, ^2^ periodontitis, ^38^ denture stomatitis, ^39^ and endodontic lesions. ^40^ We suggest that there is a synergistic relationship between the receptor for SARS-CoV-2 entry ( *ACE2*) and *C. albicans/A. actinomycetemcomitans* as the underlying mechanism of oral dysbiosis in COVID-19 patients with diabetes. These relationships may be crucial to the persistence of *C. albicans* and *A. actinomycetemcomitans* as part of the oral commensal flora and may potentially contribute to the progression of polymicrobial infection-associated dysbiosis under COVID-19 conditions.

This study has limitations. It was not possible to include all potential confounding variables. Unlike the abundance of *C. albicans*, we used the relative abundance of each targeted bacterium species as a proportion ^11^ rather than the actual levels.

## Conclusions

This study revealed that the saliva of patients with COVID-19 with diabetes retained a special relationship between SARS-CoV-2 host entry and the oral dysbiotic atmosphere represented by a unique pattern of *C. albicans* and *A. actinomycetemcomitans.* This relationship could be associated with the existence of SARS-CoV-2, but it is necessary to consider the complicity of diabetes.

## Data availability

### Underlying data

Open Science Framework: Underlying data for ‘ *ACE2* expression in saliva of patients with COVID-19 and its association with *Candida albicans* and *Aggregatibacter actinomycetemcomitans*’. https://doi.org/10.17605/OSF.IO/ENFY3 ^19^

This project contains the following underlying data:

• Data file 1: Covid-19.xlsx
• Data file 2: Ct-values.xlsx

Data are available under the terms of the Creative Commons Zero “No rights reserved” data waiver (CC0 1.0 Public domain dedication).

## Consent

Written informed consent for publication of the patients’ details was obtained from the patients.

## References

[1] Janus MM Willems HM Krom BP : Candida albicans in Multispecies Oral Communities; A Keystone Commensal?. *Adv. Exp. Med. Biol.* 2016;931:13–20. 10.1007/5584_2016_5 27271681

[2] Bachtiar EW Bachtiar BM : Relationship between Candida albicans and Streptococcus mutans in early childhood caries, evaluated by quantitative PCR. *F1000Res.* 2018;7:1645. Epub 2019/01/11. 10.12688/f1000research.16275.2 30450201 PMC6221075

[3] Thurnheer T Karygianni L Flury M : Fusobacterium Species and Subspecies Differentially Affect the Composition and Architecture of Supra- and Subgingival Biofilms Models. *Front. Microbiol.* 2019;10:1716. Epub 2019/08/17. 10.3389/fmicb.2019.01716 31417514 PMC6683768

[4] Sztukowska MN Dutton LC Delaney C : Community Development between Porphyromonas gingivalis and Candida albicans Mediated by InlJ and Als3. *MBio.* 2018;9(2). Epub 2018/04/25. 10.1128/mBio.00202-18 29691333 PMC5915736

[5] Bachtiar BB Sunarto EW Soeroso H : ACE2 gene expression and inflammatory conditions in periodontal microenvironment of COVID-19 patients with and without diabetes evaluated by qPCR. *MedRxiv [Preprint].* 2022; March 14, 2022. [accessed 2022 April 14]. 10.1101/2022.03.10.22271304:1-32

[6] Bachtiar EW Bachtiar BM : Effect of cell-free spent media prepared from Aggregatibacter actinomycetemcomitans on the growth of Candida albicans and Streptococcus mutans in co-species biofilms. *Eur. J. Oral Sci.* 2020;128(5):395–404. 10.1111/eos.12725 32808302

[7] Periasamy S Kolenbrander PE : Aggregatibacter actinomycetemcomitans builds mutualistic biofilm communities with Fusobacterium nucleatum and Veillonella species in saliva. *Infect. Immun.* 2009;77(9):3542–3551. 10.1128/IAI.00345-09 19564387 PMC2738031

[8] Bachtiar BM Fath T Widowati R : Quantification and Pathogenicity of Candida albicans in Denture-Wearing and Nondenture-Wearing Elderly. *Eur J Dent.* 2020;14(3):423–428. 10.1055/s-0040-1712779 32542630 PMC7440952

[9] Feldman M Ginsburg I Al-Quntar A : Thiazolidinedione-8 Alters Symbiotic Relationship in C. albicans-S. mutans Dual Species Biofilm. *Front. Microbiol.* 2016;7:140. 10.3389/fmicb.2016.00140 26904013 PMC4748032

[10] Belibasakis GN Ozturk VO Emingil G : Soluble triggering receptor expressed on myeloid cells 1 (sTREM-1) in gingival crevicular fluid: association with clinical and microbiologic parameters. *J. Periodontol.* 2014;85(1):204–210. 10.1902/jop.2013.130144 23659423

[11] Navidshad B Liang JB Jahromi MF : Correlation coefficients between different methods of expressing bacterial quantification using real time PCR. *Int. J. Mol. Sci.* 2012;13(2):2119–2132. 10.3390/ijms13022119 22408442 PMC3292011

[12] Alonso GC Pavarina AC Sousa TV : A quest to find good primers for gene expression analysis of Candida albicans from clinical samples. *J. Microbiol. Methods.* 2018;147:1–13. 10.1016/j.mimet.2018.02.010 29454005

[13] Nailis H Kucharikova S Ricicova M : Real-time PCR expression profiling of genes encoding potential virulence factors in Candida albicans biofilms: identification of model-dependent and -independent gene expression. *BMC Microbiol.* 2010;10:114. Epub 2010/04/20. 10.1186/1471-2180-10-114 20398368 PMC2862034

[14] Xu H Zhong L Deng J : High expression of ACE2 receptor of 2019-nCoV on the epithelial cells of oral mucosa. *Int. J. Oral Sci.* 2020;12(1):8. Epub 2020/02/26. 10.1038/s41368-020-0074-x 32094336 PMC7039956

[15] Azzi L Carcano G Gianfagna F : Saliva is a reliable tool to detect SARS-CoV-2. *J. Infect.* 2020;81(1):e45–e50. 10.1016/j.jinf.2020.04.005 32298676 PMC7194805

[16] Huang N Perez P Kato T : SARS-CoV-2 infection of the oral cavity and saliva. *Nat. Med.* 2021;27(5):892–903. 10.1038/s41591-021-01296-8 33767405 PMC8240394

[17] Wang Z Zhou J Marshall B : SARS-CoV-2 Receptor ACE2 Is Enriched in a Subpopulation of Mouse Tongue Epithelial Cells in Nongustatory Papillae but Not in Taste Buds or Embryonic Oral Epithelium. *ACS Pharmacol. Transl. Sci.* 2020;3(4):749–758. 10.1021/acsptsci.0c00062 32821883 PMC7409941

[18] Mazucanti CH Egan JM : SARS-CoV-2 disease severity and diabetes: why the connection and what is to be done?. *Immun. Ageing.* 2020;17:21. Epub 2020/07/03. 10.1186/s12979-020-00192-y 32612666 PMC7325192

[19] Bachtiar EW : ACE2 expression in saliva of patients with COVID-19 and its association with Candida albicans and Aggregatibacter actinomycetemcomitans. *Open Science Framework.* 2022. 10.17605/OSF.IO/ENFY3 PMC9445561 36112976

[20] Bachtiar EW Dewiyani S Surono Akbar SM : Inhibition of Candida albicans biofilm development by unencapsulated Enterococcus faecalis cps2. *J Dent Sci.* 2016;11(3):323–330. 10.1016/j.jds.2016.03.012 30894991 PMC6395282

[21] Ricker A Vickerman M Dongari-Bagtzoglou A : Streptococcus gordonii glucosyltransferase promotes biofilm interactions with Candida albicans. *J. Oral Microbiol.* 2014;6:6. Epub 2014/02/04. 10.3402/jom.v6.23419 24490004 PMC3907680

[22] Xu H Sobue T Thompson A : Streptococcal co-infection augments Candida pathogenicity by amplifying the mucosal inflammatory response. *Cell. Microbiol.* 2014;16(2):214–231. 10.1111/cmi.12216 24079976 PMC3956708

[23] Serrano J Lopez-Pintor RM Ramirez L : Risk factors related to oral candidiasis in patients with primary Sjogren's syndrome. *Med. Oral Patol. Oral Cir. Bucal.* 2020;25(5):e700–e705. 10.4317/medoral.23719 32683379 PMC7473438

[24] Sugio CYC Garcia A Albach T : Candida-Associated Denture Stomatitis and Murine Models: What Is the Importance and Scientific Evidence?. *J Fungi (Basel).* 2020;6(2). Epub 2020/05/28. 10.3390/jof6020070 32456172 PMC7344758

[25] Segrelles-Calvo G d SAGR Llopis-Pastor E Carrillo J : Candida spp. co-infection in COVID-19 patients with severe pneumonia: Prevalence study and associated risk factors. *Respir. Med.* 2021;188:106619. Epub 2021/09/24. 10.1016/j.rmed.2021.106619 34555702 PMC8445759

[26] Delisle MS Williamson DR Perreault MM : The clinical significance of Candida colonization of respiratory tract secretions in critically ill patients. *J. Crit. Care.* 2008;23(1):11–17. 10.1016/j.jcrc.2008.01.005 18359416

[27] Heyland D Jiang X Day AG : Serum beta-d-glucan of critically ill patients with suspected ventilator-associated pneumonia: preliminary observations. *J. Crit. Care.* 2011;26(5):536.e1–536.e9. 10.1016/j.jcrc.2011.01.002 21376516

[28] Thaweboon S Thaweboon B Nakornchai S : Salivary secretory IgA, pH, flow rates, mutans streptococci and Candida in children with rampant caries. *Southeast Asian J. Trop. Med. Public Health.* 2008;39(5):893–899. 19058586

[29] Dong L Bouey J : Public Mental Health Crisis during COVID-19 Pandemic, China. *Emerg. Infect. Dis.* 2020;26(7):1616–1618. 10.3201/eid2607.200407 32202993 PMC7323564

[30] Duan L Zhu G : Psychological interventions for people affected by the COVID-19 epidemic. *Lancet Psychiatry.* 2020;7(4):300–302. 10.1016/S2215-0366(20)30073-0 32085840 PMC7128328

[31] Granger BL : Insight into the antiadhesive effect of yeast wall protein 1 of Candida albicans. *Eukaryot. Cell.* 2012;11(6):795–805. 10.1128/EC.00026-12 22505336 PMC3370456

[32] Hoyer LL Green CB Oh SH : Discovering the secrets of the Candida albicans agglutinin-like sequence (ALS) gene family--a sticky pursuit. *Med. Mycol.* 2008;46(1):1–15. Epub 2007/09/14. 10.1080/13693780701435317 17852717 PMC2742883

[33] Bachtiar BM Theodorea CF Tahapary DL : A pilot study of red complex and three genera subgingival microbiome in periodontitis subjects with and without diabetes, evaluated by MinION platform. *F1000Res.* 2021;10:79. Epub 2021/07/28. 10.12688/f1000research.28216.4 34249333 PMC8261760

[34] Gu X Song LJ Li LX : Fusobacterium nucleatum Causes Microbial Dysbiosis and Exacerbates Visceral Hypersensitivity in a Colonization-Independent Manner. *Front. Microbiol.* 2020;11:1281. Epub 2020/08/01. 10.3389/fmicb.2020.01281 32733392 PMC7358639

[35] Takahashi Y Watanabe N Kamio N : Expression of the SARS-CoV-2 Receptor ACE2 and Proinflammatory Cytokines Induced by the Periodontopathic Bacterium Fusobacterium nucleatum in Human Respiratory Epithelial Cells. *Int. J. Mol. Sci.* 2021;22(3). Epub 2021/02/13. 10.3390/ijms22031352 33572938 PMC7866373

[36] Bachtiar EW Bachtiar BM Jarosz LM : AI-2 of Aggregatibacter actinomycetemcomitans inhibits Candida albicans biofilm formation. *Front. Cell. Infect. Microbiol.* 2014;4:94. Epub 2014/08/08. 10.3389/fcimb.2014.00094 25101248 PMC4104835

[37] Henderson B Ward JM Ready D : Aggregatibacter (Actinobacillus) actinomycetemcomitans: a triple A* periodontopathogen?. *Periodontol.* 2010;54(1):78–105. 10.1111/j.1600-0757.2009.00331.x 20712635

[38] Reynaud AH Nygaard-Ostby B Boygard GK : Yeasts in periodontal pockets. *J. Clin. Periodontol.* 2001;28(9):860–864. 10.1034/j.1600-051x.2001.028009860.x 11493356

[39] Altarawneh S Bencharit S Mendoza L : Clinical and histological findings of denture stomatitis as related to intraoral colonization patterns of Candida albicans, salivary flow, and dry mouth. *J. Prosthodont.* 2013;22(1):13–22. 10.1111/j.1532-849X.2012.00906.x 23107189 PMC3541428

[40] Baumgartner JC Watts CM Xia T : Occurrence of Candida albicans in infections of endodontic origin. *J. Endod.* 2000;26(12):695–698. 10.1097/00004770-200012000-00003 11471635

