# Supplementary material for: ACE2 expression in saliva of patients with COVID-19 and its association with Candida albicans and Aggregatibacter actinomycetemcomitans
Source: F1000Res. 2022 Sep 12;11:557. Originally published 2022 May 23. [Version 2] doi: 10.12688/f1000research.111965.2 (PMC9445561; doi:10.12688/f1000research.111965.2)
Supplement: Peer review history

# Peer review: Reviewer response for version 1

**DOI:** 10.5256/f1000research.123691.r143943

**Published:** 2022-09-05

**Authors:** Jukun Song

**Affiliations:** 1 Department of Oral and Maxillofacial Surgery, Guizhou Provincial People’s Hospital, Guiyang, China

In the manuscript titled “ACE2 expression in saliva of patients with COVID-19 and its association with Candida albicans and Aggregatibacter actinomycetemcomitans”, the authors collected 23 hospitalized patients admitted to Universitas Indonesia Hospital with PCR-confirmed COVID-19 and six healthy participants. They found that ACE2 expression can be detected in saliva of patients with COVID-19 and its association with C. albicans and gram-negative oral bacteria might contribute toward developing an oral dysbiosis based predictor for prognosis of COVID-19 severity. The study is well done.

 But there are several problems the authors should consider.

1. The study is case-control study, the sample size is too small, and statistical power lack of strength.
2. The authors demonstrated that ACE2 expression can be detected in saliva of patients with COVID-19 severity. The authors should add the association between ACE2 and COVID-19 severity.
3. In Figure 5A, The AUC is 1, and the results were ideal.

Is the work clearly and accurately presented and does it cite the current literature?

Yes

If applicable, is the statistical analysis and its interpretation appropriate?

Yes

Are all the source data underlying the results available to ensure full reproducibility?

Yes

Is the study design appropriate and is the work technically sound?

Partly

Are the conclusions drawn adequately supported by the results?

Yes

Are sufficient details of methods and analysis provided to allow replication by others?

Partly

Reviewer Expertise:

I am committed to the mechanism research of oral and maxillofacial tumors.

I confirm that I have read this submission and believe that I have an appropriate level of expertise to confirm that it is of an acceptable scientific standard.

# Response

**Published:** 2022-09-06

**Authors:** Endang Bachtiar

**RC**: The study is case-control study, the sample size is too small, and statistical power lack of strength.

**AR**: Thank you for your suggestion. As suggested, we included this suggestion in the paragraph of limitation, as follows:

 “This study has limitations. It was not possible to include all potential confounding variables. Unlike the abundance of *C. albicans*, we used the relative abundance of each targeted bacterium species as a proportion [10] rather than the actual levels. Moreover, due to the sample size is too small, the statistical power might lack of power.

**RC**: The authors demonstrated that ACE2 expression can be detected in saliva of patients with COVID-19 severity. The authors should add the association between ACE2 and COVID-19 severity.

**AR**: Thank you for this important suggestion. However, in this study we did not have any data regarding the COVID-19 severity, as we have mentioned in Discussion section (paragraph 3, line 2), as follows:

 “According to the medical records, all patients had received a non-invasive supplemental device. However, we did not have any data regarding how long the patients had received the device at the time the samples were collected”.

**RC**: In Figure 5A, The AUC is 1, and the results were ideal.

**AR**: Thank you for your positive comment.

# Peer review: Reviewer response for version 1

**DOI:** 10.5256/f1000research.123691.r138780

**Published:** 2022-08-08

**Authors:** Juni Handajani

**Affiliations:** 1 Department of Oral Biology, Faculty of Dentistry, Universitas Gadjah Mada, Yogyakarta, Indonesia

The topic of this research is interesting and the research method used is appropriate. The results of this study are useful to determine the mechanism of the relationship between the entry of the SARS-CoV-2 host with the oral dysbiotic atmosphere. In this study, *C. albicans* and actinomycetemcomitans were used. I would like to suggest adding more descriptions on the use of *A. actinomycetemcomitans* in the background. In order to know the clinical oral health of the subject, could you please add the oral condition data to the results? Data on the condition of the oral cavity of the subject can be used to discuss the relationship between bacteria and fungi in the dysbiotic atmosphere of the oral cavity. Also could you please add the number of subjects (n) in Figures 1B and 1C.

Is the work clearly and accurately presented and does it cite the current literature?

Yes

If applicable, is the statistical analysis and its interpretation appropriate?

Yes

Are all the source data underlying the results available to ensure full reproducibility?

Yes

Is the study design appropriate and is the work technically sound?

Yes

Are the conclusions drawn adequately supported by the results?

Yes

Are sufficient details of methods and analysis provided to allow replication by others?

Yes

Reviewer Expertise:

Oral Biology

I confirm that I have read this submission and believe that I have an appropriate level of expertise to confirm that it is of an acceptable scientific standard.

# Response

**Published:** 2022-09-06

**Authors:** Endang Bachtiar

Reviewer’s comment ( **RC**): I would like to suggest adding more descriptions on the use of A. actinomycetemcomitans in the background.

 Author response **(AR): **Thank you for this important suggestion. As suggested,  we added more description regarding *A. actinomycetemcomitans*, in the last paragraph of the background, as follow:

 “Therefore,  our objective was to investigate the level of C. albicans, its pathogenicity, and to evaluate its antagonistic relationship with *Aggregatibacter actinomycetemcomitans* [5], which is a keystone pathogen associated with periodontitis in adolescents [6], in the saliva of COVID-19 patients with and without diabetes. We included Fusobacterium nucleatum and *Veillonella parvula *as their relationship with *Aggregatibacter actinomycetemcomitans* has been previously reported [7]”.

**RC**: In order to know the clinical oral health of the subject, could you please add the oral condition data to the results?

**AR**: Thank you for your suggestion. We have added the oral health condition in discussion section, not in the results section. It can be seen in paragraph 3, line 5, as follows:

 “Additionally, most participants had poor oral hygiene (not shown), and we did not measure the local risk factors. However, the high count of salivary *C. albicans* observed may indicate that low salivary flow rate influences salivary colonization by this fungus [26]”,…….

**RC**: Also, could you please add the number of subjects (n) in Figures 1B and 1C.

**AR**: Thank you for your suggestion. We have revised the caption of Figures 1, as follows:

 Fig. 1. Standard curves and melt curves. Standard curve of *C. albicans* ATCC 10231 (A) and representative melt curve graph for *C. albicans* count in saliva  (B) and tongue surface (C) **in all participants**.

 The bold fond indicated the added word.
